# Supplementary material for: Low expression of RNA sensors impacts Zika virus infection in the lower female reproductive tract
Source: Nat Commun. 2019 Sep 25;10:4344. doi: 10.1038/s41467-019-12371-7 (PMC6761111; doi:10.1038/s41467-019-12371-7)
Supplement: Supplementary file 1 — Supplementary Information [file 41467_2019_12371_MOESM1_ESM.pdf]

Supplementary Information

Low expression of RNA sensors impacts Zika virus infection in the lower female reproductive tract

Khan et al.

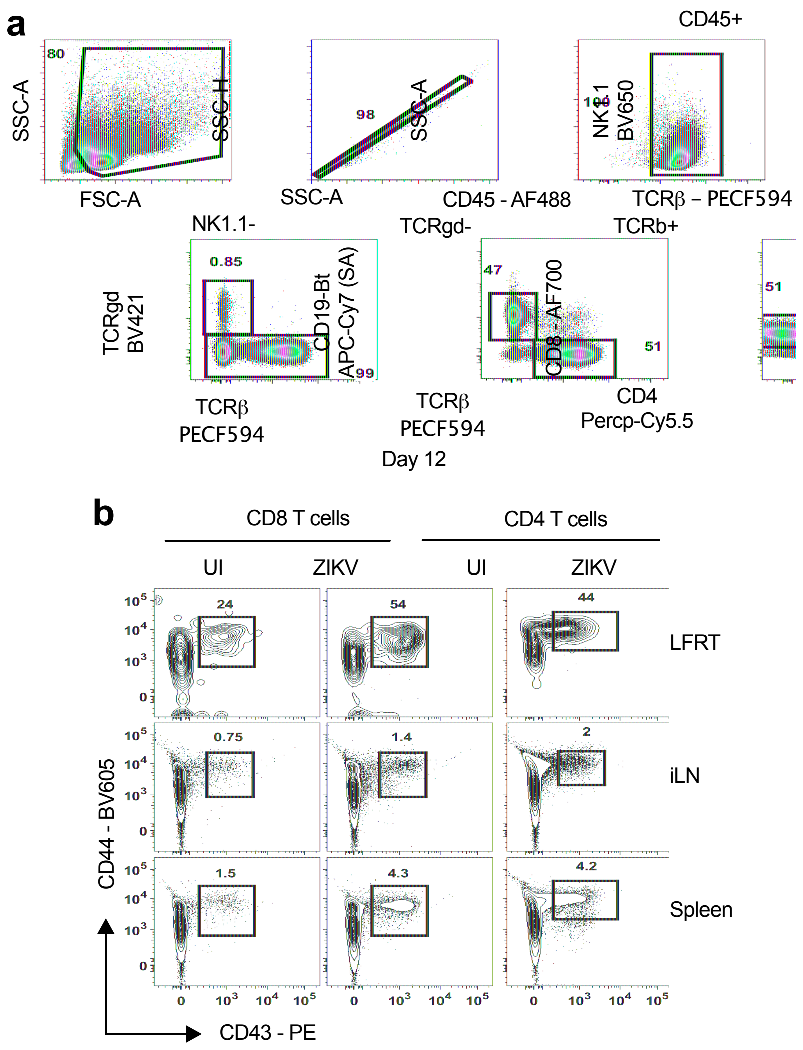

Supplementary Figure 1: Vaginal ZIKV infection enhances the number and activation of T cells.

Related to Figure 1. Groups of DMPA-treated female mice were i.vag. inoculated with 2×10^{4} FFU of ZIKV (PRVABC59). (a) Gating strategy for identifying different immune cell populations. (b) Representative ratio of activated T cells in indicated tissues in UI and ZIKV infected mice at day 12 p.i..

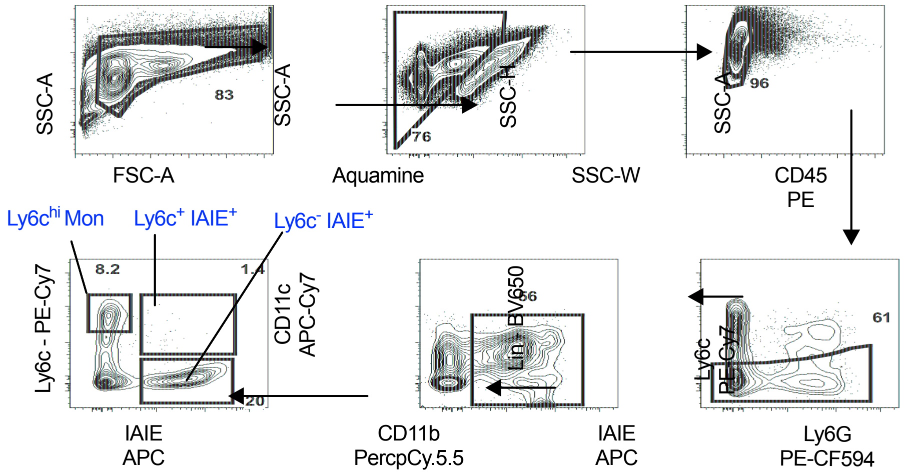

Supplementary Figure 2: Gating strategy to identify CD11b^{+} monocyte populations.

Related to Figure 2. Groups of DMPA-treated female mice were i.vag. inoculated with 2×10^{4} FFU of ZIKV (PRVABC59). Representative flow plots for identifying monocyte subsets. Total cells were gated on SSC-A and FSC-A. Dead cells were excluded based on Aquamine Live/dead staining. Single cells were gated on SSC-H and SSC-W. Hematopoietic cells were identified based on CD45 staining. After excluding Ly6c^{+}Ly6G^{+} neutrophils and Lin+ (TCRβ^{+} NK1.1^{+} CD19^{+} EpCAM-1^{+}) cells, CD11b^{+} cells were divided into three different monocyte populations: 1) Ly6c^{hi} monocytes, 2) Ly6c^{+} IAIE^{+} cells, and 3) Ly6c^{-} IAIE^{+} cells. LFRT, lower female reproductive tract.

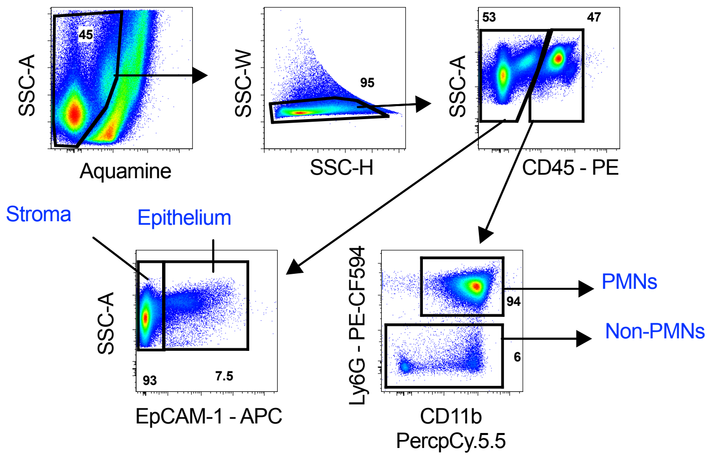

Supplementary Figure 3: Gating strategy for LFRT cell sorting.

Related to Figure 6. Live hematopoietic and non-hematopoietic cells from LFRT were sorted by first excluding doublets based on SSC-W and SSC-H gating. The $CD45^{-}$ cells were further divided into EpCAM-1^{+} epithelial and EpCAM-1^{-} stromal cells. The $CD45^{+}$ cells were then gated on $CD11b^{+}$ $Ly6G^{+}$ polymorphonuclear neutrophils (PMNs) or other $Ly6G^{-}$ hematopoietic cells.

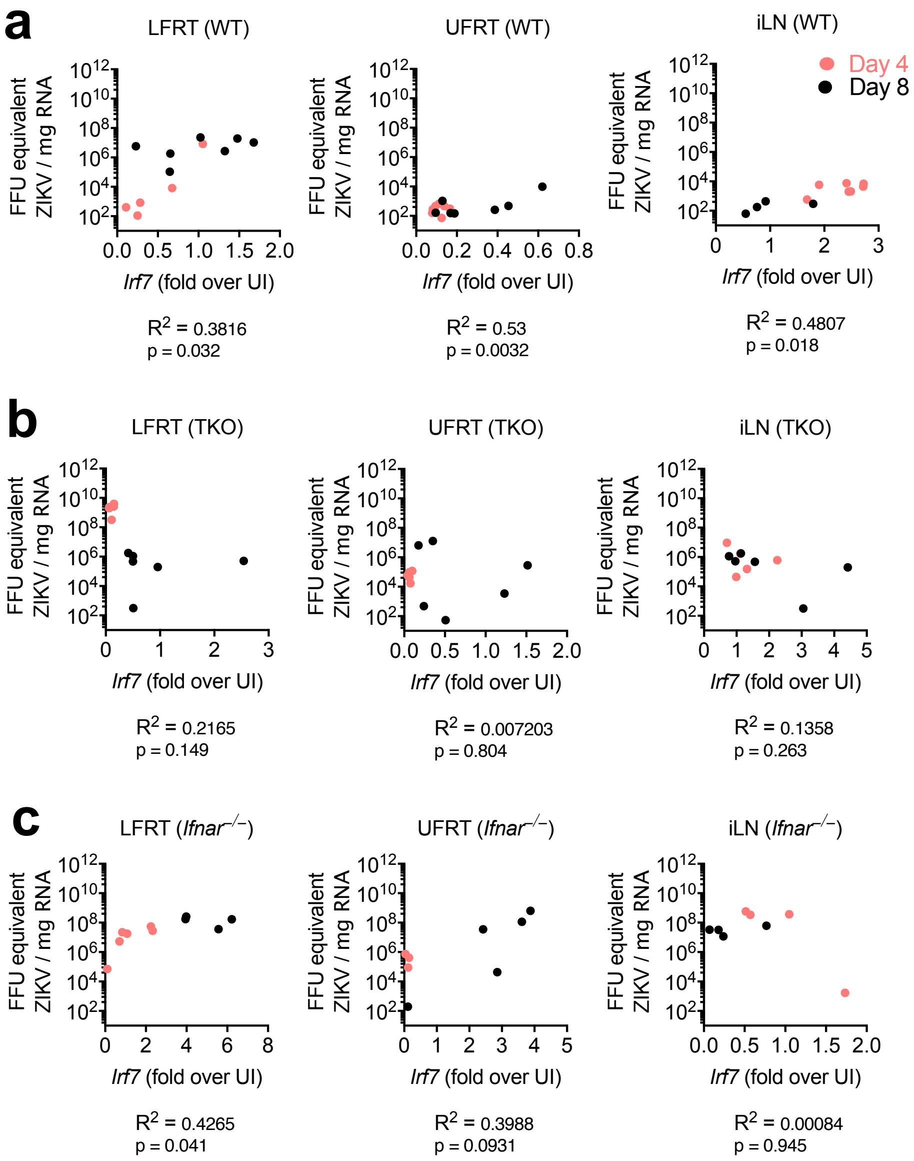

Supplementary Figure 4: Linear regression analysis of ZIKV copy numbers and *Irf7* induction.

Related to Figure 7. Groups of DMPA-treated (a) WT, (b) TKO, or (c) *Ifnar1*$^{-/-}$ female mice were i.vag. inoculated with 2 × 10^4 FFU of ZIKV (PRVABC59). At days 4 and 8 post i.vag. infection, ZIKV RNA copies and *Irf7* fold induction were measured in indicated tissues (as also shown in Fig. 6) and the data were re-plotted here for linear regression analysis. Source data for (a-c) are provided as a Source Data file.

Supplementary Table 1: Primer sequences for qRT-PCR analyses.

**Gene**	**Forward (SYBR)**	**Reverse (SYBR)**	**Accession no.**
ZIKV	GAGACGAGATGCGGTACAGG	CGACCGTCAGTTGAACTCCA	
Gapdh (Macaque)	GGAGTCAACGGATTTGGTCG	ATGACGAGCTTCCCGTTCTC	NM_001195426.1
Tlr3 (Macaque)	CCTGGTTTGTTAATTGGATTAACGA	TGAGGTGGAGTGTTGCAAAGG	XM_015139554.1
Tlr7 (Macaque)	TTGGCACCTCTCATGCTCTG	GTGTCCACACTGGAAACATCATT	NM_001130426.2
Ddx58 (Macaque)	GCTGATGAAGGCATTGACATTGC	CAGAAGGAAGCACTTGCTACCTC	NM_001042668.1
Ifih1 (Macaque)	CTGACTTGCCCTCTCCATCG	GCAGCAATCCGGTTTCTGTCT	NM_001047123.2
Mex3b (Macaque)	TAAAATCAAAGCGCTGCGGG	TGTTCTTATTCCGGGAGGCG	XM_001110186.3
Irf7 (Macaque)	GGAGAGGGACAAGAGGGTCT	TGGCCTCGCCTGTCATTAG	NM_001136100.1
Gapdh (Mouse)	TGTGTCCGTCGTGGATCTGA	CCTGCTTCACCACCTTCTTGA	NM_008084.3
Tlr3 (Mouse)	CTCTTGAACAACGCCCAACT	GTCCACTTCAGCCCAGAGAA	NM_126166.5
Tlr7 (Mouse)	ATGTGGACACGGAAGAGACAA	GGTAAGGGTAAGATTGGTGGTG	NM_133211.4
Ddx58 (Mouse)	TTGCTGAGTGCAATCTCGTC	GTATGCGGTGAACCGTCTTT	NM_172689.3
Ifih1 (Mouse)	GGCACCATGGGAAGTGATT	ATTTGGTAAGGCCTGAGCTG	NM_027835.3
Mex3b (Mouse)	ACCCAGTTCCGAACACGTCG	TGTTCTTGTTACGAGAGGCT	NM_175366.3
Irf7 (Mouse)	CAGCAGCAGTCTCGGCTTGTG	TGACCCAGGTCCATGAGGAAGTG	NM_016850.3
